# Impact of Acute Kidney Injury on the COVID-19 In-Hospital Mortality in Octogenarian Patients: Insights from the COVOCA Study

**DOI:** 10.3390/life14010086

**Published:** 2024-01-04

**Authors:** Alfredo Caturano, Raffaele Galiero, Erica Vetrano, Giulia Medicamento, Maria Alfano, Domenico Beccia, Chiara Brin, Sara Colantuoni, Jessica Di Salvo, Raffaella Epifani, Riccardo Nevola, Raffaele Marfella, Celestino Sardu, Carmine Coppola, Ferdinando Scarano, Paolo Maggi, Cecilia Calabrese, Pellegrino De Lucia Sposito, Carolina Rescigno, Costanza Sbreglia, Fiorentino Fraganza, Roberto Parrella, Annamaria Romano, Giosuele Calabria, Benedetto Polverino, Antonio Pagano, Fabio Giuliano Numis, Carolina Bologna, Mariagrazia Nunziata, Vincenzo Esposito, Nicola Coppola, Nicola Maturo, Rodolfo Nasti, Pierpaolo Di Micco, Alessandro Perrella, Luigi Elio Adinolfi, Marina Di Domenico, Marcellino Monda, Vincenzo Russo, Roberto Ruggiero, Giovanni Docimo, Luca Rinaldi, Ferdinando Carlo Sasso

**Affiliations:** 1 Department of Advanced Medical and Surgical Sciences, University of Campania “Luigi Vanvitelli”, Piazza L. Miraglia 2, 80138 Naples, Italy; raffaele.galiero@unicampania.it (R.G.); erica.vetrano@unicampania.it (E.V.); giulia.medicamento@unicampania.it (G.M.); maria.alfano2@libero.it (M.A.); domenico.beccia@unicampania.it (D.B.); chiara.brin@gmail.com (C.B.); sara.colantuoni@yahoo.it (S.C.); jessicagiulia.disalvo@unicampania.it (J.D.S.); ellaphane@gmail.com (R.E.); riccardo.nevola@unicampania.it (R.N.); raffaele.marfella@unicampania.it (R.M.); celestino.sardu@unicampania.it (C.S.); luigielio.adinolfi@unicampania.it (L.E.A.); ferdinandocarlo.sasso@unicampania.it (F.C.S.); 2 Department of Experimental Medicine, University of Campania “Luigi Vanvitelli”, 80138 Naples, Italy; marcellino.monda@unicampania.it; 3 Ospedale Evangelico Betania, 80147 Naples, Italy; 4 Hepatology Unit, Internal Medicine, Area Stabiese Hospital, 80053 Naples, Italy; carmine.coppola@aslnapoli3sud.it; 5 COVID Center “S. Anna e SS. Madonna della Neve” Hospital, 80042 Boscotrecase, Italy; ferdi.scarano@gmail.com; 6 U.O.C. Infectious and Tropical Diseases, S. Anna e S. Sebastiano Hospital, 81100 Caserta, Italy; paolo.maggi@unicampania.it; 7 U.O.C. Pneumologia Vanvitelli, Department of Translational Medical Sciences, University of Campania “Luigi Vanvitelli”, AORN Ospedali dei Colli, Via Leonardo Bianchi, 80131 Naples, Italy; cecilia.calabrese@unicampania.it; 8 COVID Center, Maddaloni Hospital, 80124 Maddaloni, Italy; pellegrino.delucia@aslcaserta1.it; 9 U.O.C. Infectious Diseases and Neurology, Cotugno Hospital, 80131 Naples, Italy; carolina.rescigno@ospedalideicolli.it; 10 U.O.C. Infectious Diseases of the Elderly, Cotugno Hospital, 80131 Naples, Italy; costanza.sbreglia@ospedalideicolli.it; 11 U.O.C. Anestesia and Intensive Care Unit, Cotugno Hospital, 80131 Naples, Italy; fiorentino.fraganza@ospedalideicolli.it; 12 U.O.C. Respiratory Infectious Diseases, Cotugno Hospital, 80131 Naples, Italy; roberto.parrella@ospedalideicolli.it; 13 U.O.C. Pneumology, Moscati Hospital, 83100 Avellino, Italy; annareromano@gmail.com; 14 IXth Division of Infectious Diseases and Interventional Ultrasound, Cotugno Hospital, 80131 Naples, Italy; g.calabria@tin.it; 15 “Giovanni da Procida” Hospital, 84126 Salerno, Italy; benedetto.polverino@sangiovannieruggi.it; 16 Emergency and Acceptance Unit, “Santa Maria delle Grazie” Hospital, 80078 Pozzuoli, Italy; antoniopag82@gmail.com (A.P.); fabiogiuliano.numis@aslnapoli2nord.it (F.G.N.); 17 Internal Medicine Unit, Ospedale Del Mare, 80147 Naples, Italy; carolina.bologna@libero.it; 18 U.O.C. Internal Medicine, Moscati Hospital, 83100 Avellino, Italy; mg.nunziata@gmail.com; 19 IVth Division of Immunodeficiency and Gender Infectious Diseases, Cotugno Hospital, 80131 Naples, Italy; vincenzoesposito@ospedalideicolli.it; 20 COVID Center, Department of Mental Health and Public Medicine, A.O.U. Vanvitelli, 80131 Naples, Italy; nicola.coppola@unicampania.it; 21 U.O.S.D. Infectious Diseases Emergency and Acceptance, Cotugno Hospital, 80131 Naples, Italy; nicola.maturo@ospedalideicolli.it; 22 Emergency Division, A.O.R.N. “Antonio Cardarelli”, 80131 Naples, Italy; rodolfo.nasti@gmail.com; 23 Department of Internal Medicine, Fatebenefratelli Hospital of Naples, 80123 Naples, Italy; pdimicco@libero.it; 24 Task Force COVID-19 Regione Campania, 80131 Napoli, Italy; alessandro.perrella@aocardarelli.it; 25 Department of Precision Medicine, University of Campania “Luigi Vanvitelli”, 80131 Naples, Italy; marina.didomenico@unicampania.it; 26 Department of Biology, College of Science and Technology, Sbarro Institute for Cancer Research and Molecular Medicine, Temple University, Philadelphia, PA 19122, USA; v.p.russo@libero.it; 27 Division of Cardiology, Department of Medical Translational Sciences, University of Campania “Luigi Vanvitelli”, 80131 Naples, Italy; 28 Division of General, Oncological, Mini-Invasive and Obesity Surgery, University of Campania “Luigi Vanvitelli”, 80131 Naples, Italy; roberto.ruggiero@unicampania.it; 29 Unit of Thyroid Surgery, Department of Medical and Advanced Surgical Sciences, University of Campania “Luigi Vanvitelli”, 80138 Naples, Italy; giovanni.docimo@unicampania.it; 30 Department of Medicine and Health Sciences “Vincenzo Tiberio”, University of Molise, 86100 Campobasso, Italy; luca.rinaldi@unicampania.it

**Keywords:** acute kidney injury, octogenarian, COVID-19, in-hospital mortality, SARS-CoV-2

## Abstract

Background and Aims: The COVID-19 pandemic, caused by the novel coronavirus SARS-CoV-2, has fundamentally reshaped the landscape of global public health, with some people suffering more adverse clinical outcomes than others. The aim of this study is to deepen our understanding of the specific impact of acute kidney injury (AKI) on the in-hospital mortality in octogenarian patients with COVID-19. Methods: This is a prospective observational cohort study, which involved 23 COVID-19 hospital units in the Campania Region, Italy. Exposure variables were collected during hospital admission and at discharge. Only patients aged ≥80 years were deemed eligible for the study. Results: 197 patients were included in the study (median age 83.0 [82.0–87.0] years; 51.5% men), with a median duration of hospitalization of 15.0 [8.0–25.0] days. From the multivariable Cox regression analysis, after the application of Šidák correction, only the respiratory rate (HR 1.09, 95% CI: 1.04 to 1.14; *p* < 0.001) and AKI development (HR: 3.40, 95% CI: 1.80 to 6.40; *p* < 0.001) were independently associated with the primary outcome. Moreover, the Kaplan–Meier analysis showed a significantly different risk of in-hospital mortality between patients with and without AKI (log-rank: <0.0001). Conclusions: In our investigation, we identified a significant association between AKI and mortality rates among octogenarian patients admitted for COVID-19. These findings raise notable concerns and emphasize the imperative for vigilant monitoring of this demographic cohort.

## 1. Introduction

The COVID-19 pandemic, caused by the novel coronavirus SARS-CoV-2, has fundamentally reshaped the landscape of global public health. This highly transmissible virus manifests with diverse clinical presentations, ranging from mild respiratory symptoms to severe systemic complications [1,2]. It has been reported by the World Health Organization that the global COVID-19 pandemic has resulted in a total mortality of 6,981,263 deaths [3]. As our understanding of COVID-19 has evolved, it has become increasingly evident that certain demographic groups face unique challenges, and among them, octogenarian individuals have emerged as a particularly vulnerable population [4,5]. In fact, frailty, identified as a geriatric condition, is characterized by physiological alterations affecting the musculoskeletal, neuroendocrine, and immune systems [6]. These changes not only contribute to an accelerated trajectory of functional decline but also intersect with a myriad of comorbidities commonly observed in octogenarians, including cardiovascular diseases, diabetes, and compromised immune function. This convergence of factors renders octogenarians more susceptible to severe health outcomes [6,7].

Recent epidemiological reports have underscored the elevated mortality rates and heightened risk of complications among octogenarians diagnosed with COVID-19 [7]. Additionally, individuals with pre-existing chronic kidney disease (CKD) may face heightened risks and challenges when infected with COVID-19. In addition, COVID-19 can exacerbate existing renal conditions, leading to a decline in kidney function [8]. Acute kidney injury (AKI), a critical renal complication, has garnered increased attention in the context of COVID-19. The virus’s impact on renal health extends beyond exacerbating pre-existing conditions, with emerging evidence suggesting a direct link between COVID-19 infection and the development of AKI. This raises concerns about the potential implications of AKI for patient outcomes, especially in vulnerable populations such as octogenarians [9]. Despite the recognized importance of renal health in the context of COVID-19, the specific consequences of AKI for certain demographic groups, especially octogenarians, remain less explored. The aim of this study is to deepen our understanding of the specific impact of AKI on in-hospital mortality in octogenarian patients with COVID-19.

## 2. Methods and Materials

### 2.1. Study Design and Participants

COVOCA (observational study on the COVID-19 population hOspitalized in the CAmpania Region) is a prospective observational cohort study that engaged 23 COVID-19 centers within hospitals across the Campania Region, Italy. The study centered on adult patients (≥18 years old) hospitalized due to SARS-CoV-2 infection between 1 November 2020 and 30 June 2021. Individuals with missing or incomplete laboratory and clinical data at the commencement or conclusion of their hospitalization were excluded from the study. The primary sources of data for the study comprised electronic records and clinical charts of each hospitalized subject. From the COVOCA dataset of 1403 hospitalized subjects with a positive swab for SARS-CoV-2, 209 individuals aged ≥80 years were deemed eligible for the study. Following the exclusion of 12 patients due to missing data, a total of 197 patients were included in the present analysis. All patients discharged alive were phone-called to confirm their 30-day survival. All patients provided written informed consent. The study received approval from the local Ethics Committees (Universita’ degli studi della Campania “Luigi Vanvitelli”, Azienda Ospedaliera Universitaria, “Luigi Vanvitelli”, and Azienda Ospedaliera di Rilievo Nazionale “Ospedali dei colli”; ID 10879/I; approval date: 11 May 2020) and aligned with the principles outlined in the 1976 Declaration of Helsinki and its subsequent amendments.

### 2.2. Variables (Outcome and Exposure)

The diagnosis of SARS-CoV-2 infection was established through real-time polymerase chain reaction (RT-PCR) analysis of specimens obtained via nasal–pharyngeal swabs. In assessing in-hospital mortality and length of stay, either death certificates or discharging letters were utilized. Upon admission and discharge of subjects, details of the following exposure variables were collected: (a) Anthropometric and demographic characteristics; (b) Anamnestic data, incorporating the number of vaccinated individuals, type of vaccine received, COVID-19 positive cases in the family, and the duration between diagnosis and hospitalization; (c) Symptoms and signs experienced by patients, including cough, anosmia, fever, diarrhea, chest and abdominal pain, dysgeusia (including the onset day), dyspnea, and altered consciousness; (d) Information on pre-existing comorbidities, such as diabetes, smoking habits, chronic cardiac disease, hypertension, chronic liver disease (CLD), chronic kidney disease (CKD), chronic respiratory disease, cancers, and chronic neurological disorders; (e) Details about drugs administered at the beginning and during hospitalization for infection treatment; (f) Regarding laboratory data collected for the COVOCA registry, specific attention was directed to creatinine, and the estimated glomerular filtration rate (eGFR) was calculated using the CKD-Epi formula.

Diabetes mellitus was identified following the guidelines outlined by the American Diabetes Association (ADA). The diagnosis was derived from anamnestic records, coupled with laboratory examinations performed upon admission [10]. In a similar manner, hypertension diagnosis adhered to the guidelines set by the European Society of Hypertension and the European Society of Cardiology, supplemented by anamnestic data [11]. Chronic cardiac conditions, including heart failure, previous acute myocardial infarction (AMI), ischemic cardiopathy, atrial fibrillation, and valvulopathy, were diagnosed based on medical history and clinical investigation. For other conditions such as chronic liver disease (CLD), chronic respiratory diseases, chronic kidney disease (CKD), malignancies, and neurologic disorders, the diagnosis relied on the anamnesis of each subject. 

AKI was diagnosed according to KDIGO guidelines: (1) an increase in serum creatinine of ≥0.3 mg/dL (≥26.5 µmol/L) within 48 h; (2) an increase in serum creatinine to ≥1.5 times the baseline within the previous 7 days; (3) urine volume ≤ 0.5 mL/kg/h for 6 h [12].

### 2.3. Statistical Analysis

Categorical data were summarized as frequencies in absolute and relative percentages. Continuous variables were expressed as either the mean and standard deviation (SD) or the median and interquartile range (IQR), depending on their distribution, as assessed using the Shapiro–Wilk test. Regarding missing data, categorical variables were categorized as ”Missing”, representing a specific category for each variable. For continuous variables, no imputation methods were applied, and missing information was denoted as not applicable (N/A) in the dataset.

Population data were stratified into two groups based on AKI development during the in-hospital stay. Group differences were assessed using *p* values, calculated through ANOVA or Kruskal–Wallis tests for continuous data and chi-squared or Fisher’s exact tests for categorical data, depending on the distribution and sample size. We considered *p* values less than 5% as statistically significant in our analyses. Additionally, a logistic regression analysis was conducted on the medications administered during the in-hospital stay to investigate whether they could be associated with the development of AKI.

Our primary endpoint, in-hospital mortality, was evaluated using Kaplan–Meier survival analysis, with log-rank tests to compare survival curves between patients with or without AKI. The Kaplan–Meier curve further allowed us to visually depict survival estimates over time. The follow-up time lasted from hospitalization until the discharge (or death) date to ensure inclusion of all enrolled patients.

To identify potential prognostic factors influencing survival outcomes, univariable and multivariable Cox proportional hazards regression models were utilized. These models provided hazard ratios (HR) and their corresponding 95% confidence intervals (CI), assessing the strength and significance of factors in relation to the primary endpoint. Šidák correction was applied to address the issue of multiple testings in the context of multivariable Cox regression analysis. Šidák correction was used to counteract the problem of multiple comparisons (α < 0.007).

All statistical analyses were performed using RStudio (RStudio Team (2016); RStudio: Integrated Development for R. RStudio, Inc., Boston, MA, USA; URL http://www.rstudio.com/, accessed on 3 August 2023).

## 3. Results

We included 197 patients with positive swabs for SARS-CoV-2 in the study (median age 83.0 [82.0–87.0] years; 51.5% men) followed at our referral centers for a median hospitalization period of 15.0 [8.0–25.0] days. Throughout the observation period, 91 patients (53.1%) experienced a primary outcome event; 38 (74.5%) in the AKI subgroup and 53 (37.1%) in the non-AKI group (*p* < 0.001). Of note, the causes of mortality included 48 cases (52.7%) due to ARDS, 17 cases (18.7%) due to septic shock, 3 cases (3.3%) due to acute coronary syndrome, 1 case (1.1%) related to complications of multiple myeloma, 1 case (1.1%) following diarrhea from clostridium difficile, 18 cases (19.8%) of sudden death, and 3 cases (3.3%) due to ab ingestis pneumonia. The AKI group exhibited a higher prevalence of moderate/severe impaired consciousness, along with lower levels of eGFR and a shorter duration of hospitalization. All discharged patients were alive 30 days after discharge. All the baseline clinical characteristics of the study population are summarized in Table 1. 

From the univariate logistic regression analysis, diuretics demonstrated a significant association with the development of AKI (OR 5.76, 95% CI 2.79 to 11.90; *p* < 0.001). No other drugs showed a significant association with AKI in this analysis.

From the multivariable Cox regression analysis (Table 2), after the application of Šidák correction, only respiratory rate (HR 1.09, 95% CI 1.04 to 1.14; *p* < 0.001) and AKI development (HR: 3.40, 95% CI 1.80 to 6.40; *p* < 0.001) were independently associated with the primary outcome.

**Figure 1:**
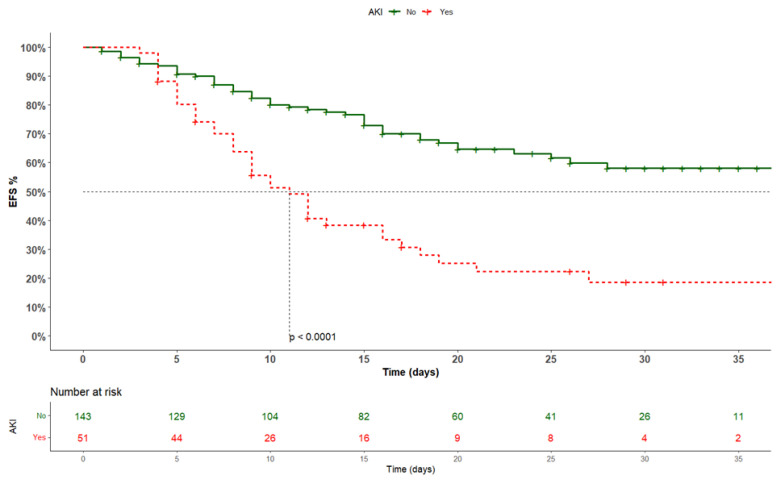
Kaplan–Meier analysis according to AKI development. AKI: acute kidney injury; EFS: event-free survival.

The Kaplan–Meier analysis showed a significantly different risk of the primary outcome event between the two subgroups (log-rank: <0.0001) (Figure 1).

**Figure 2:**
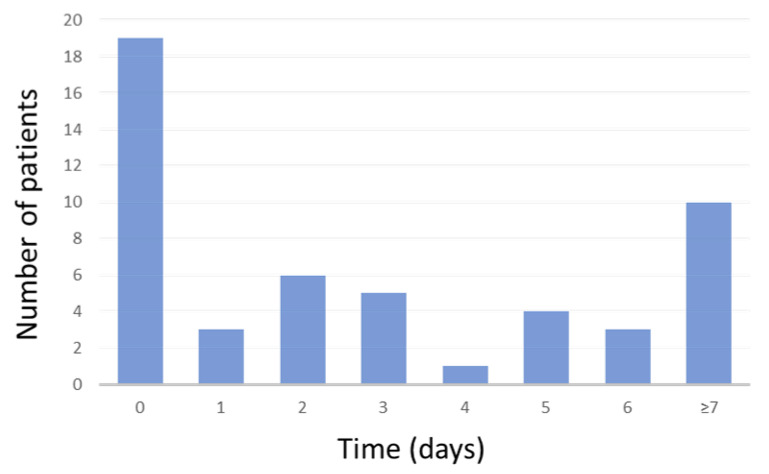
Time to AKI development in our cohort over days of hospitalization.

In 19 cases of AKI (37.3%), this was already present at in-hospital admission. The time to AKI development in our cohort study is shown in Figure 2.

## 4. Discussion

The main finding of the current study demonstrates that AKI development in COVID-19 octogenarian patients seems to be a predictor of in-hospital mortality. In addition, we also report an AKI incidence of 26.3%, which was mostly already present at in-hospital admission.

AKI frequently complicates ARDS, arising from factors such as impaired oxygenation, fluid overload, cardiogenic shock, sepsis, or injurious mechanical ventilation [13]. In the context of COVID-19, the incidence of AKI has been observed in a range of 0.5% to 39% [14,15,16,17,18], similar to our findings. Despite its widespread occurrence, the exact pathophysiology of AKI in COVID-19 continues to be a subject of ongoing research. The multifaceted impact of the SARS-CoV-2 virus on the renal system is predominantly facilitated through the angiotensin-converting enzyme 2 (ACE2) pathway. The virus’s spike protein binds to ACE2 receptors in host cells, initiating a cascade of events that can result in diverse renal conditions [19]. Through this pathway, several pathological outcomes have been observed, including acute tubular necrosis, protein leakage in Bowman’s capsule, collapsing glomerulopathy, and mitochondrial impairment [20]. The direct impact of SARS-CoV-2 on renal cells is underscored by studies such as that by Puelles et al., which detected the virus in various kidney compartments. The evidence of RNA enrichment for ACE2 in diverse kidney cell types supports the hypothesis of a direct viral influence on renal cells [21]. Cytopathic effects on these cells, as evidenced by autopsy findings revealing acute tubular necrosis and inflammatory cell infiltration, further substantiate the intricate relationship between the virus and renal manifestations in COVID-19 [22,23]. The dysregulation of immune responses, triggered by SARS-CoV-2, indirectly contributes to AKI. In particular, cytokine storm, macrophage activation syndrome, and lymphopenia are among the immune-mediated mechanisms associated with renal damage. In fact, the release of various inflammatory mediators, such as interleukin (IL)-6, IL-1β, tumor necrosis factor-alpha, inducible protein-10, monocyte chemotactic protein 1, granulocyte-colony stimulating factor, and macrophage inflammatory protein-1α, can directly contribute to kidney injury through the resultant activation of the innate immune system [24,25,26]. This occurs against the backdrop of the hypotension associated with a cytokine storm and the superimposed sepsis, further elevating the risk of AKI [24]. The intricate interplay of these immune responses amplifies the complexity of renal involvement in COVID-19. Beyond immune dysregulation, other potential mechanisms of AKI have been identified. These include organ interactions, endothelial dysfunction, hypercoagulability, rhabdomyolysis, and sepsis. Additionally, a decrease in oxygen delivery to the kidneys may lead to ischemic injury, further exacerbating renal complications in COVID-19 patients [24,27]. Finally, COVID-19 treatment has included different drug therapies operating through distinct mechanisms, with certain medications carrying nephrotoxic implications [28,29,30]. Hydroxychloroquine, largely used during the first wave in Italy [31,32] and antiviral agents, including lopinavir, ritonavir, and remdesivir, also pose a potential risk of kidney injury [28,29], with documented instances of renal impairment reported in users of remdesivir [33]. Furthermore, intravenous immunoglobulin, in addition to antivirals, carries a potential risk of proximal tubular injury [28,29]. Although the underlying pathophysiology varies, the incidence of AKI in COVID-19 has consistently shown a strong association with increased mortality, as evident in both our data and the existing literature [34]. In our study, we have observed an association between diuretic use and the heightened risk of AKI, which aligns with the pharmacological effects of diuretics on renal physiology. Diuretics, by design, enhance urine production and electrolyte excretion to alleviate fluid overload and manage hypertension. However, this intensified diuresis can lead to intravascular volume depletion and subsequent reductions in renal blood flow, potentially compromising kidney function [12]. Our findings are consistent with those reported in a recent meta-analysis [9], emphasizing the importance for clinicians to be cautious about the potential nephrotoxic effects of diuretics, despite the absence of an association with the primary outcome.

A recent meta-analysis also revealed that advanced age independently increased the risk of AKI in COVID-19 patients, with an odds ratio of 3.53 [16]. In elderly patients infected with SARS-CoV-2, the increased mortality rates suggest a potential link to weakened immune system function and the aging of tissues, rendering them more susceptible to viral replication. This observation aligns with the broader context of age-related vulnerabilities, where the aging process contributes to physiological changes that may compromise the body’s defense mechanisms [35,36]. Intriguingly, this age-related susceptibility contrasts sharply with observations in the pediatric population, where, despite having an impaired immune system during infancy, children tend to exhibit less severe symptoms of COVID-19 [37]. It has been reported that older adults generally exhibit a reduction in ACE2-positive cells, primarily localized in the lower pulmonary tract, coupled with a concurrent decrease in lung progenitor cells, potentially influencing the severity of the disease and the recuperation process from pneumonia resulting from SARS-CoV-2 infection in elderly individuals [38]. Moreover, even in the absence of statistical differences in the number of days of infection before hospitalization, the significant incidence of AKI at admission (10%) may imply a more aggressive manifestation of the disease in these patients. Indeed, a recent meta-analysis reported that the occurrence of AKI is more than five times higher in severe cases and non-survivors compared to non-severe cases and survivors [39]. Likewise, this observation might partially substantiate the independent association between the respiratory rate at admission and adverse clinical outcomes, as revealed by the COX regression multivariate analysis. Unfortunately, the parameter of respiratory rate remains relatively underexplored in the context of COVID-19 patients. Specifically, in a recent meta-analysis, respiratory rate was identified as a risk factor in only a limited number of studies; specifically, five in total. Regrettably, amalgamation of the data and the derivation of risk estimates encountered challenges due to the inherent heterogeneity stemming from the disparate reported outcomes and risk measures [40].

While our study contributes to the existing body of evidence, it also highlights the ongoing need for research in this field. Further investigations into the specific mechanisms linking AKI to in-hospital mortality, the long-term consequences of renal complications, and the efficacy of targeted therapeutic interventions are crucial. Collaborative efforts across research institutions may accelerate the pace of discovery and contribute to more effective strategies for managing AKI in the context of COVID-19.

The present study is subject to several limitations that warrant consideration. Firstly, while our study included a substantial number of participants, the sample size may still limit the generalizability of our findings to broader populations. Larger, more diverse cohorts are necessary to validate and extend the external validity of our results. Secondly, a limitation arises from the lack of data collection throughout the entire hospitalization period spanning from admission to discharge. Such comprehensive data collection would have been instrumental in identifying forms of AKI that manifested within the initial 48 h of hospitalization, offering a more detailed temporal perspective. Thirdly, the absence of body mass index (BMI) and related data presents another limitation. The inclusion of BMI as a variable in the analysis could have contributed to a more nuanced understanding of its potential association with AKI in the context of COVID-19. Fourthly, the study lacks pre-hospital admission creatinine values. Inclusion of these values would have provided valuable baseline information regarding the patients’ kidney function before the onset of the infection, offering insights into the dynamics of renal changes during the course of COVID-19. Fifthly, the timeframe of enrollment predates the widespread vaccination campaign against SARS-CoV-2 in Italy. Consequently, the study lacks complete data on the vaccination status of participants, preventing the incorporation of this variable into the statistical analysis. This limitation highlights the evolving nature of the pandemic and the need for ongoing research to capture the impact of vaccination on COVID-19 outcomes. Lastly, there was an absence of laboratory data on urinary protein levels, sediment measurements, and hematuria, which could have provided valuable insights into the phenotype of renal damage, thus enhancing our understanding of the renal implications of COVID-19 [41]. In addition, the absence of biopsy data restricts our ability to elucidate the specific histological renal damage that occurred in COVID-19 patients. While such data would have been valuable for a comprehensive understanding, the emergency status induced by the pandemic and the characteristics of the patients made it logistically challenging to perform renal biopsies during the study period.

## 5. Conclusions

In our investigation, we identified a significant association between AKI and mortality rates among octogenarian patients admitted with COVID-19. These findings raise notable concerns and emphasize the imperative for vigilant monitoring of this demographic cohort.

## Figures and Tables

**Table 1:** Baseline characteristics of the study population presented as overall data and stratified according to AKI development.

Parameter	Overall (n = 194)	Non-AKI Development (n = 143)	AKI Development (n = 51)	*p*
**Age**, median [IQR]	83.0 [82.0–87.0]	83.0 [81.3–87.0]	84.0 [82.0–87.0]	0.562
**Sex**, n (%)				
M	100 (51.5)	70 (49.0)	30 (58.8)	0.207
F	94 (48.5)	73 (51.0)	21 (41.2)	
**Duration of hospitalization**, median [IQR]	15.0 [8.0–25.0]	16.0 [9.0–25.0]	10.0 [6.0–16.8]	**0.007**
**Days before hospitalization**, median [IQR]	6.0 [3.0–9.0]	5.0 [3.0–8.0]	7.0 [1.3–12.8]	0.688
**Body temp (°C)**, median [IQR]	36.3 [36.0–37.0]	36.3 [36.0–37.0]	36.3 [36.0–37.1]	0.956
**History of fever,** n (%)	109 (56.2)	81 (56.6)	28 (54.9)	0.830
**Respiratory rate (apm)**, median [IQR]	20.0 [18.0–25.0]	20.0 [16.8–25.0]	22.0 [18.0–28.0]	0.056
**Heart rate (bpm)**, median [IQR]	80.0 [73.0–93.0]	80.0 [73.0–90.0]	85.5 [73.5–100.0]	0.255
**Blood pressure (mmHg)**, median [IQR]				
Systolic	135.0 [120.0–145.0]	135.0 [120.0–145.0]	130.0 [120.0–153.0]	0.928
Diastolic	73.5 [69.0–80.0]	70.0 [66.0–80.0]	77.5 [70.0–80.0]	0.217
**Diarrhea**, n (%)	15 (7.7)	12 (8.4)	3 (5.9)	0.566
**Oxygen saturation %**, median [IQR]	93.0 [88.0–96.0]	93.0 [89.0–96.0]	94.0 [87.0–97.0]	0.566
**GCS/15**, n (%)				
Mild/non-impaired consciousness	167 (86.1)	128 (89.5)	39 (76.5)	**0.021**
Moderate/Severe impaired consciousness	27 (13.9)	15 (10.5)	12 (23.5)	
**Oxygen therapy**, n (%)	99 (51.0)	70 (48.9)	29 (56.9)	0.134
**Chronic cardiac disease**, n (%)	92 (49.5)	63 (44.1)	29 (56.9)	0.053
**CKD**, n (%)	37 (18.8)	23 (16.1)	14 (27.5)	0.097
**eGFR, mL/min/1.73 m**^2^, median [IQR]	59.1 [34.7–75.7]	63.0 [46.4–80.6]	34.6 [20.4–57.7]	**<0.001**
**Hypertension**, n (%)	147 (75.8)	108 (73.5)	39 (83.0)	0.187
**Diabetes**, n (%)	50 (25.8)	35 (23.8)	15 (31.9)	0.223
**Smoking**, n (%)	15 (7.7)	14 (9.5)	1 (2.1)	0.099
**CLD**, n (%)	12 (6.2)	10 (7.0)	2 (3.9)	0.436
**Chronic Respiratory Disease**, n (%)	51 (26.3)	34 (23.8)	17 (33.3)	0.184
**Chronic neurological disorder**, n (%)	42 (21.6)	32 (22.4)	10 (19.6)	0.681
**Malign**, n (%)	24 (12.4)	16 (10.9)	8 (17.0)	0.267
**In-hospital mortality**, n (%)	91 (53.1)	53 (37.1)	38 (74.5)	**<0.001**
**In-hospital Drugs**				
**Steroids**, n (%)	175 (90.2)	131 (91.6)	44 (86.3)	0.209
**Monoclonal Abs**, n (%)	2 (1.0)	1 (0.7)	1 (2.0)	0.445
**Antivirals**, n (%)	25 (12.9)	21 (14.7)	4 (7.8)	0.212
**Antibiotics**, n (%)	166 (87.4)	122 (85.3)	44 (86.2)	0.784
**NSAIDs,** n (%)	29 (14.9)	24 (16.8)	5 (9.8)	0.244
**Anticoagulants,** n (%)	188 (96.9)	137 (95.8)	51 (100)	0.138
**Diuretics, n (%)**	82 (42.3)	45 (31.4)	37 (72.5)	**<0.001**

Abbreviations: M: male; F: female; IQR: interquartile range; apm: acts per minute; bpm: beats per minute; Body temp: body temperature; GCS: Glasgow Coma Score; CKD: chronic kidney disease; CLD: chronic liver disease; Malign: malignancies; NSAID: nonsteroidal anti-inflammatory drug.

**Table 2:** Univariable and multivariable Cox’s regression model for the primary outcome among the study population.

	Univariable Analysis				Multivariable Analysis			
Parameter	HR	95% CI		*p*	HR	95% CI		*p*
**Age**	1.01	0.96	1.06	0.637				
**Sex**								
M (ref)	1							
F	0.86	0.56	1.30	0.464				
**Days before hospitalization**	1.03	0.92	1.14	0.650				
**Body temp (°C)**	1.11	0.86	1.12	0.429				
**History of fever**	0.93	0.61	1.41	0.724				
**Respiratory rate (apm)**	1.12	1.07	1.18	**<0.001**	1.09	1.04	1.14	**<0.001**
**Blood pressure (mmHg)**								
Systolic blood pressure	0.98	0.97	0.99	**0.004**	0.98	0.97	0.99	**0.022**
Diastolic blood pressure	0.99	0.97	1.01	0.205				
**Diarrhea**	0.40	0.13	1.28	0.123				
**Heart rate (bpm)**	1.02	1.01	1.03	**0.017**	1.00	0.99	1.02	0.161
**Oxygen saturation**	0.96	0.94	0.98	**<0.001**	0.96	0.94	0.99	0.015
**GCS**	0.62	0.36	1.05	0.077				
**Oxygen therapy**	1.34	0.89	2.03	0.162				
**Chronic cardiac disease**	1.67	1.08	2.58	**0.020**	1.38	0.76	2.50	0.295
**Hypertension**	0.85	0.53	1.35	0.483				
**CKD**	1.38	0.84	2.26	0.203				
**Diabetes**	1.01	0.62	1.63	0.974				
**Smoking**	0.38	0.12	1.21	0.103				
**CLD**	0.51	0.16	1.60	0.248				
**Chronic respiratory disease**	1.22	0.77	1.93	0.388				
**Chronic neurological disorder**	0.87	0.52	1.43	0.574				
**Malign**	0.92	0.47	1.78	0.800				
**Steroids**	0.73	0.36	1.46	0.373				
**Antivirals**	0.51	0.24	1.10	0.087				
**Antibiotics**	1.07	0.69	1.68	0.757				
**NSAIDs**	0.42	0.19	0.93	**0.031**	0.79	0.24	2.60	0.704
**Diuretics**	1.84	1.19	2.82	**0.006**	0.79	0.42	1.49	0.470
**Anticoagulants**	0.39	0.14	1.07	0.068				
**AKI development**	2.60	1.70	3.97	**<0.001**	3.96	1.87	8.41	**<0.001**
**eGFR**	0.99	0.98	0.998	**0.015**	1.01	0.99	1.02	0.278

Abbreviations: HR: hazard ratio; M: male; F: female; GCS: Glasgow Coma Score; CKD: chronic kidney disease; NSAID: nonsteroidal anti-inflammatory drug.

## Data Availability

The data that support the findings of this study are available on reasonable request from the corresponding author.
